# Molecular surveillance of foodborne bacterial pathogens and resistome in food products from Hong Kong

**DOI:** 10.1099/mic.0.001661

**Published:** 2026-02-02

**Authors:** Qiao Hu, Lianwei Ye, Tao Zang, Chen Yang, Xuemei Yang, Ruanyang Sun, Edward Wai Chi Chan, Sheng Chen

**Affiliations:** 1 Department of Infectious Diseases and Public Health, Jockey Club College of Veterinary Medicine and Life Sciences, City University of Hong Kong, Kowloon, Hong Kong SAR; 2 State Key Lab of Chemical Biology and Drug Discovery and the Department of Food Science and Nutrition, The Hong Kong Polytechnic University, Hung Hom, PR China; 3 Shenzhen Key Lab for Biological Safety Control, The Hong Kong Polytechnic University Shenzhen Research Institute, Shenzhen, PR China

**Keywords:** food products, genomics, pathogen isolation, plasmid characterization, resistome profiles

## Abstract

Foodborne infections pose an increasing public health challenge worldwide. The problem has been aggravated by the dissemination of antimicrobial resistance genes among zoonotic pathogens, which results in a sharp increase in antibiotic resistance rate recorded among the major foodborne pathogens. To obtain an overview of the extent to which food products purchased in the markets in Hong Kong were contaminated by foodborne pathogens, we collected 95 raw meat samples from wet markets and isolated 236 bacterial strains of various species, with *Escherichia coli* being the most dominant species (131 strains). Contamination of food products by multiple foodborne pathogens was commonly observed. These include both Gram-positive and Gram-negative bacteria that exhibit various levels of resistance, with some possessing multiple clinically important antibiotic resistance genes. Seventeen bacterial strains of various species isolated from three food samples were comprehensively analysed by the Oxford Nanopore R10.4 technology. Novel conjugative plasmids carrying antimicrobial resistance gene-bearing mobile genetic elements were commonly detectable in the test strains. Some of the plasmids were shown to have originated from other environmental sources or other bacterial species, indicating that raw foods in the local market may serve as a reservoir of resistance-encoding genetic elements from which such elements are disseminated to various microbial pathogens. These findings suggest a need to perform periodic but comprehensive surveillance of multidrug-resistant bacterial pathogens and the major antimicrobial resistance genes in common food products, so as to disrupt the transmission routes of such organisms and the resistance-encoding genetic elements that they harbour.

## Availability of Data and Materials

The whole-genome sequencing, including individual accession numbers for each strain (SAMN40576005, SAMN40576004, SAMN40576003, SAMN40576002, SAMN40576001, SAMN40576000, SAMN40575999, SAMN40575998, SAMN40575997, SAMN40575996, SAMN40575995, SAMN40575994, SAMN40575993, SAMN40575992, SAMN40575991, SAMN4057590 and SAMN40575989), has been submitted to NCBI under BioProject accession number PRJNA1090847.

## Introduction

Food safety has become a major public health concern in recent years, prompting government policy makers, clinicians and researchers to devise effective approaches to prevent and treat foodborne microbial infections. The surveillance data in the USA estimated that 31 major pathogens caused a total of 9.4 million episodes of foodborne illness each year, resulting in 55,961 hospitalizations and 1,351 deaths [1]. Among these foodborne infections, seafood-associated bacterial gastroenteritis was mainly caused by *Vibrio parahaemolyticus* [2,3]. The ubiquitous spore-forming bacterium *Bacillus cereus* is known to cause widespread food poisoning incidents [4]. Detection of infectious agents such as *Salmonella* spp. and *Staphylococcus aureus* in various common food products is also increasingly being described, suggesting that food samples are a key reservoir of various opportunistic bacterial pathogens that may cause food poisoning [5,6]. Through the years, novel synthetic compounds, natural products, peptides and proteins have been exhaustively studied in order to identify novel agents that can be used to combat foodborne pathogens such as *Escherichia coli* [7]. However, the development and dissemination of phenotypic antimicrobial resistance (AMR) among bacterial pathogens that reside in food samples, different environmental niches and clinical settings have severely compromised efforts to control bacterial infections. Plasmids carrying AMR-encoding genes (ARGs) render bacteria to evolve efficiently into multidrug-resistant (MDR) strains, which pose a severe threat to animal and human health [8,9]. A variety of mobile resistance and/or virulence genes are now detectable in food animals, meat, manure and various environmental and wastewater samples; hence, the risk by which these genetic elements are transmitted to humans via the food chain has increased significantly. One example is carbapenem-resistant *Enterobacteriaceae* (CRE) which has been recognized as a cause of difficult-to-treat infections associated with high mortality, particularly those which occur in health-care facilities. Resistance development in CRE has been attributed to excessive clinical use of β-lactams of different generations including penicillin, cephalosporins, carbapenems and monobactams [10,12]. Although many efforts have been devoted to the development of new antibiotics and non-traditional antibacterial compounds, few new drugs are available for treatment of nosocomial infections caused by MDR Gram-negative bacterial pathogens, a number of which have been classified as critical priority pathogens by WHO [13,15].

This study aims to investigate the epidemiological characteristics of potential bacterial pathogens, as well as their resistance phenotypes, by performing a surveillance of meat products purchased from a wet market in Hong Kong, followed by analysis of their phenotypic and genetic traits with the focus on the potential transmission of different AMR bacterial pathogens and their resistance determinants in the same food sample. Data obtained in this work should facilitate the development of effective food safety policy and intervention strategies to minimize the chance of occurrence of foodborne infections.

## Methods

### Bacterial isolation

The sampling locations represented typical wet markets in Kowloon, the New Territories (including both village-based markets and urban town centres) and Hong Kong Island. Samples were collected between May and November 2022, covering late spring to autumn, when ambient temperature may influence bacterial contamination levels. Five types of fresh meat, including beef (20), chicken (15), pork (25), freshwater fish (15) and shrimp (20), were collected and transported to the laboratory immediately (Table 1). The sample size (95 raw meat samples) provides a snapshot rather than population-level estimates. The different numbers of food types reflected availability and sales volume in local wet markets during sampling, rather than deliberate stratified design. Briefly, 5 g of each sample was aseptically transferred to 45 ml of PBS buffer, followed by homogenization and incubation in enriching broth for 18–24 h; the culture was then inoculated onto LB agar plates. A total of five bacterial species were studied: *S. aureus*, *B. cereus*, *Salmonella* spp., *E. coli* and *Vibrio* spp. Enrichment broths for different pathogens were processed in parallel rather than sequentially to avoid competitive exclusion. Selective media containing antibiotics of breakpoint concentration were applied following standard workflows (XLT4 for *Salmonella*, Baird-Parker agar for *S. aureus*, TCBS for *Vibrio* and MYP for *B. cereus*). Only one colony of typical morphology was picked from each plate for downstream analyses; therefore, within-sample strain diversity may be underestimated. After purification, the strains were identified by MALDI-TOF MS using a Bruker UltrafleXtreme MALDI-TOF/TOF MS. Bacterial strains were preserved in glycerol stock solution (20% v/v) and stored at −80 °C for subsequent genetic and phenotypic characterization.

**Table 1.** The number and proportion of different types of food samples collected in different regions of Hong Kong in this study

No. of sample	Kowloon	New Territories	Hong Kong Island	Total
Beef	10	7	3	20
Chicken	6	6	3	15
Pork	9	11	5	25
Fish	8	6	1	15
Shrimp	7	9	4	20

### Antimicrobial susceptibility test

The minimal inhibitory concentrations (MICs) of the foodborne isolates were determined by the agar dilution, as well as the broth microdilution method according to protocols of the Clinical and Laboratory Standards Institute (CLSI). Representative antibiotics of β-lactams, aminoglycosides and fluoroquinolones were tested: cefotaxime, meropenem, gentamicin, tigecycline, ciprofloxacin, vancomycin, erythromycin, colistin, azithromycin, clindamycin, aztreonam, ceftazidime and tetracycline. Resistance breakpoints were interpreted according to CLSI recommendations. *E. coli* ATCC 25922 and *S. aureus* ATCC 29213 were used as the quality control strains.

### Nanopore sequencing and bioinformatic analyses

Three samples (pork-12, pork-13 and fish-1) were selected as they contained the highest number of co-existing bacterial species and exhibited multidrug resistance, making them suitable for investigating plasmid structures and potential ARG exchange. The selection aimed to maximize genomic diversity rather than represent all food types. A total of 17 strains of various species recovered from 3 food samples (pork-12, pork-13 and fish-1) were subjected to nanopore sequencing. Genomic DNAs were extracted by using QIAamp DNA Kits (QIAGEN, Inc., Netherlands) according to the manufacturer’s protocols. The DNA concentration and purity were quantified with the Qubit fluorometer and Thermo Scientific Nanodrop spectrophotometer, respectively.

For sequencing in the MinION platform (Oxford Nanopore Technologies, Oxford, UK)[16], the library was prepared using the Rapid Barcoding Kit 96 V14 (SQK-RBK114.96), following the manufacturer’s protocols. Sequencing was then performed on the MinION platform using the R10.4.1 flow cell, and the procedure was allowed to run for 72 h. The MinKNOW software was applied to collect raw sequencing data from MinION. POD5 files were base-called using Dorado (Oxford Nanopore Technologies) and then subjected to barcode trimming with Guppy. Alignment of the plasmids and nanopore long reads was visualized by the Easyfig tools [17]. ARGs were identified using Abricate (version 1.0.0) (Seemann)[18] against the Comprehensive Antibiotic Resistance Database [19] and NCBI AMRFinderPlus [20].

## Results

### Prevalence of foodborne opportunistic pathogens in 95 raw meat samples

**Fig. 1.**
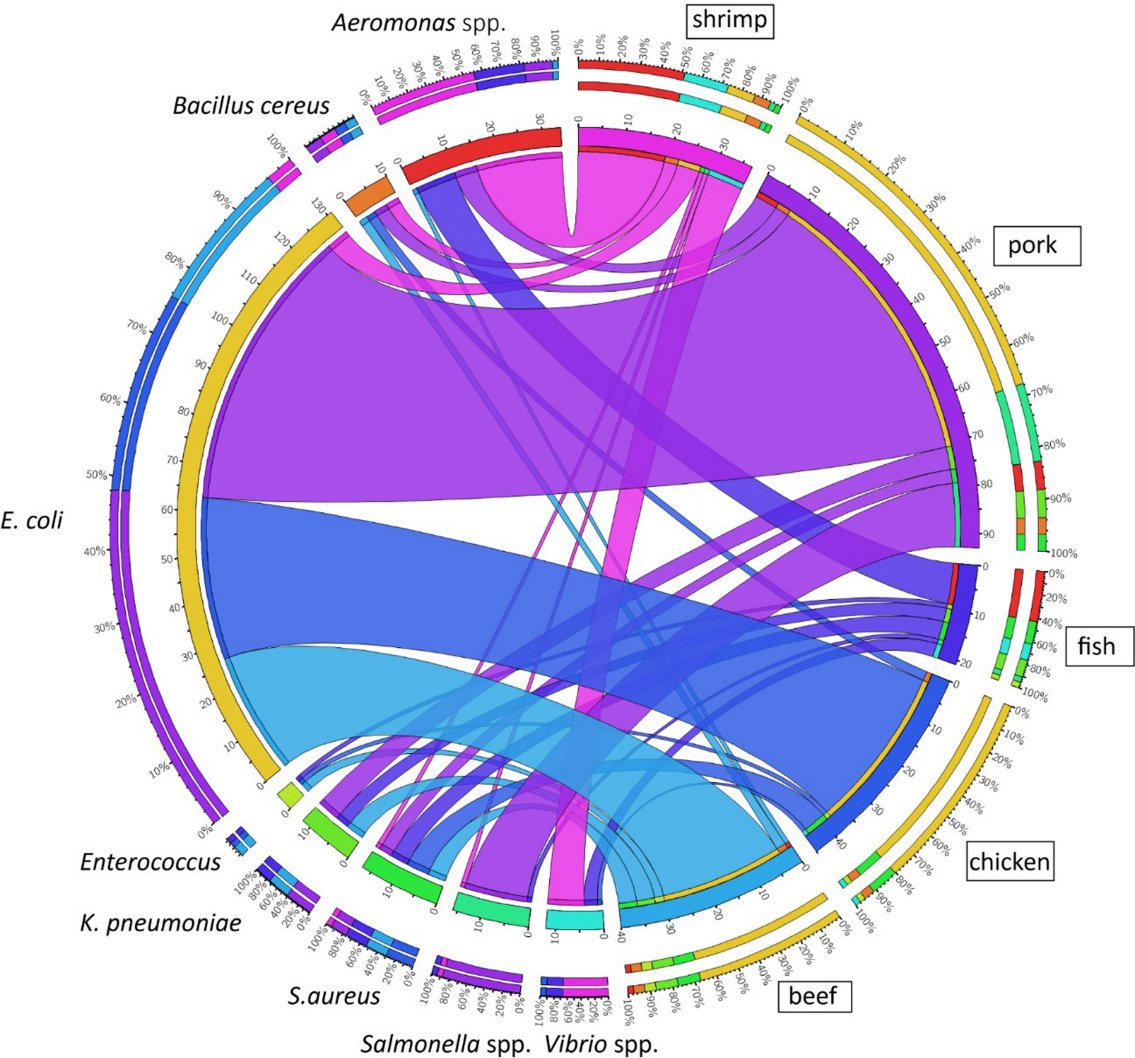
The relative abundance of bacterial strains of various species recovered from different types of meat samples. Visualization by Circos [42]: the right half consists of five categories of meat samples, and the left half consists of strains of eight groups of bacterial species recovered from each food type. The degree of association between each food sample and different bacterial species is shown.

A total of 236 bacterial strains were isolated from 95 raw meat samples (Table 1). There were 131 *E. coli* strains detectable in 50 samples except fish; 16 *Salmonella* spp. isolates were recovered from 9 samples, 17 *S. aureus* strains were isolated from 16 samples, and 12 *Vibrio* spp. isolates were recovered from 8 samples (Table 2, Fig. 1). The rate of recovery of *B. cereus* was relatively low, as only 10 strains could be isolated from 6 of the 95 meat samples. In addition to the target organisms that we intended to investigate in the original study plan, other bacterial species including 34 *Aeromonas* spp., 12 *Klebsiella pneumoniae* and 4 *Enterococcus faecalis* strains were persistently recoverable from various selective media. *E. coli* was the most abundant species of all the isolates, with almost half (63 out of 131) originating from pork. Notably, pork was the food product from which the majority of *Salmonella enterica* were isolated. On the other hand, although it is commonly regarded as a poultry-associated bacteria, *Salmonella* could not be recovered in the 15 chicken samples. Of all the 236 meat-borne strains, pork was the most predominant source of bacterial contamination with a total of 93 bacterial strains being isolated from 24 pork samples. Among them, *E. coli* isolates were found in 23 pork samples, and 1 such sample, pork-12, was found to be contaminated with 4 different species of bacteria, namely *S. aureus* (1), *K. pneumoniae* (1), *E. coli* (2) and *S. enterica* (4). Another sample, pork-13, was found to be contaminated with three *E. coli* and three *S. enterica* strains. Moreover, the 15 raw chicken samples were found to harbour a total of 45 bacterial isolates, the majority of which were *E. coli* (36) and *S. aureus* (5). Furthermore, 40 strains were recovered from beef samples, 37 from shrimp samples and 21 from 7 freshwater fish samples. Like pork and chicken, the main organism recovered from beef was *E. coli* (27), but other bacterial species were also detectable, including four *S. aureus* strains, four *K. pneumoniae* strains and two each of *B. cereus* and *E. faecalis*. In aquatic products such as shrimps and freshwater fishes, *Aeromonas* spp. (19 and 9, respectively) was the most prevalent species, followed by strains of the *Vibrio* spp. (8 and 3, respectively).

**Table 2.** The types of bacterial strains recovered from different food samples in this study

No. of strains	Beef	Chicken	Pork	Fish	Shrimp	Total
*E. coli*	27 (12)	36 (12)	63 (23)	0	5 (3)	131
*Aeromonas*	1 (1)	0	5 (5)	9 (6)	19 (12)	34
*Salmonella*	0	0	14 (7)	1 (1)	1 (1)	16
*B. cereus*	2 (1)	2 (1)	3 (2)	0	3 (2)	10
*S. aureus*	4 (4)	5 (4)	3 (3)	4 (4)	1 (1)	17
*K. pneumoniae*	4 (4)	0	5 (4)	3 (2)	0	12
*Vibrio*	0	1 (1)	0	3 (3)	8 (4)	12
*E. faecalis*	2 (1)	1 (1)	0	1 (1)	0	4
Total	40	45	93	21	37	236

(), number of samples with microbiological contaminants. *E. coli*, *S. aureus* and *B. cereus* were confidently identified to species level by MALDI-TOF. Some isolates (e.g. *Aeromonas*, *Salmonella *spp. and *Vibrio *spp.) were reported only at the genus level when MALDI-TOF confidence scores did not meet the species-level threshold.

### Phenotypic AMR characteristics of meat-borne bacterial strains

Analysis of MICs of 131 *E. coli* strains showed that the AMR rate of these strains was very high, with about half of the strains (64 out of 131 and 66 out of 131) being resistant towards cefotaxime and ciprofloxacin, respectively. Resistance to meropenem and tigecycline was relatively low, accounting for 13% (17 out of 131) and 2% (3 out of 131) of the *E. coli* strains, respectively. Colistin resistance (MIC ≥4 mg l^−1^, broth microdilution) was detected in 12.2% (16 out of 131) of *E. coli* isolates. However, another 108 strains also exhibited intermediate resistance to this antibiotic (Table 3). Among the five types of meats, chicken-borne *E. coli* strains exhibited the highest rate of resistance to cefotaxime, meropenem and ciprofloxacin. The 27 beef-derived *E. coli* strains exhibited a higher rate of resistance towards ciprofloxacin than the other 2 β-lactam drugs (CTX, MRP), as did the 63 strains recovered from pork samples. On the other hand, the 16 *Salmonella* strains displayed the highest rate of resistance towards ciprofloxacin [37.5% (6 out of 16)], followed by 18.8% (3 out of 16), 6.25% (1 out of 16) and 6.25% (1 out of 16), to tigecycline, meropenem and cefotaxime, respectively. Furthermore, *Aeromonas* spp. displayed the highest rate of resistance to tetracycline and cefotaxime (22 out of 33 or 66.7%). A lower level of AMR was observed among the *Vibrio* spp. and *B. cereus* strains, with 33.3% (4 out of 12) of *Vibrio* strains being cefotaxime resistant, and 20% (2 out of 10) *B. cereus* isolates exhibiting ciprofloxacin resistance. Importantly, the 12 *K*. *pneumoniae* strains recovered in this work were resistant to multiple antibiotics, with cefotaxime and tetracycline being the least effective drugs in case these *K. pneumoniae* strains cause foodborne infections. Furthermore, one strain recovered from a fish sample was resistant to all test antibiotics except tigecycline (Table 3). All in all, our data showed that the foodborne bacteria were mainly susceptible to meropenem and tigecycline but exhibited different levels of resistance to other antimicrobial agents.

**Table 3.** AMR profiles of foodborne bacterial isolates of different species

Rate of resistance	MRP	CTX	CAZ	TIG	TET	CIP	GEN	ATM	CST
*E. coli*	17/131 (13.0%)	64/131 (48.9%)	11/44 (25.0%)	1/72 (1.4%)	n/a	66/131 (50.4%)	18/44 (40.9%)	n/a	16/131 (12.2%)
*K. pneumoniae*	3/12 (25.0%)	7/12 (58.3%)	4/12 (33.3%)	0/12	10/12 (83.3%)	n/a	3/12 (25.0%)	4/12 (33.3%)	3/12 (25.0%)
*Aeromonas* spp.	0/33	22/33 (66.7%)	15/33 (45.5%)	2/33 (6.1%)	22/33 (66.7%)	5/33 (15.1%)	1/33 (3.0%)	6/33 (18.2%)	n/a
*Salmonella* spp.	1/16 (6.2%)	3/16 (18.7%)	n/a	1/16 (6.2%)	n/a	6/16 (37.5%)	n/a	n/a	4/16 (25.0%)
*Vibrio* spp.	0/12	4/12 (33.3%)	n/a	0/12	3/12 (25.0%)	2/12 (16.7%)	n/a	n/a	n/a
*B. cereus*	1/10 (10.0%)	n/a	n/a	0/10	n/a	2/10 (20.0%)	0/10	n/a	n/a

n/a, the test was not conducted. n/a indicates that the antibiotic was not tested for that species. Not all species were tested against the full antibiotic panel because CLSI interpretive criteria were unavailable or the test was not relevant for that organism.

ATM, aztreonam; CAZ, ceftazidime; CIP, ciprofloxacin; CST, colistin; CTX, cefotaxime; GEN, gentamicin; MRP, meropenem; TET, tetracycline; TIG, tigecycline.

### Genetic characteristics of bacterial strains of bacteria isolated from the same food sample

**Fig. 2.**
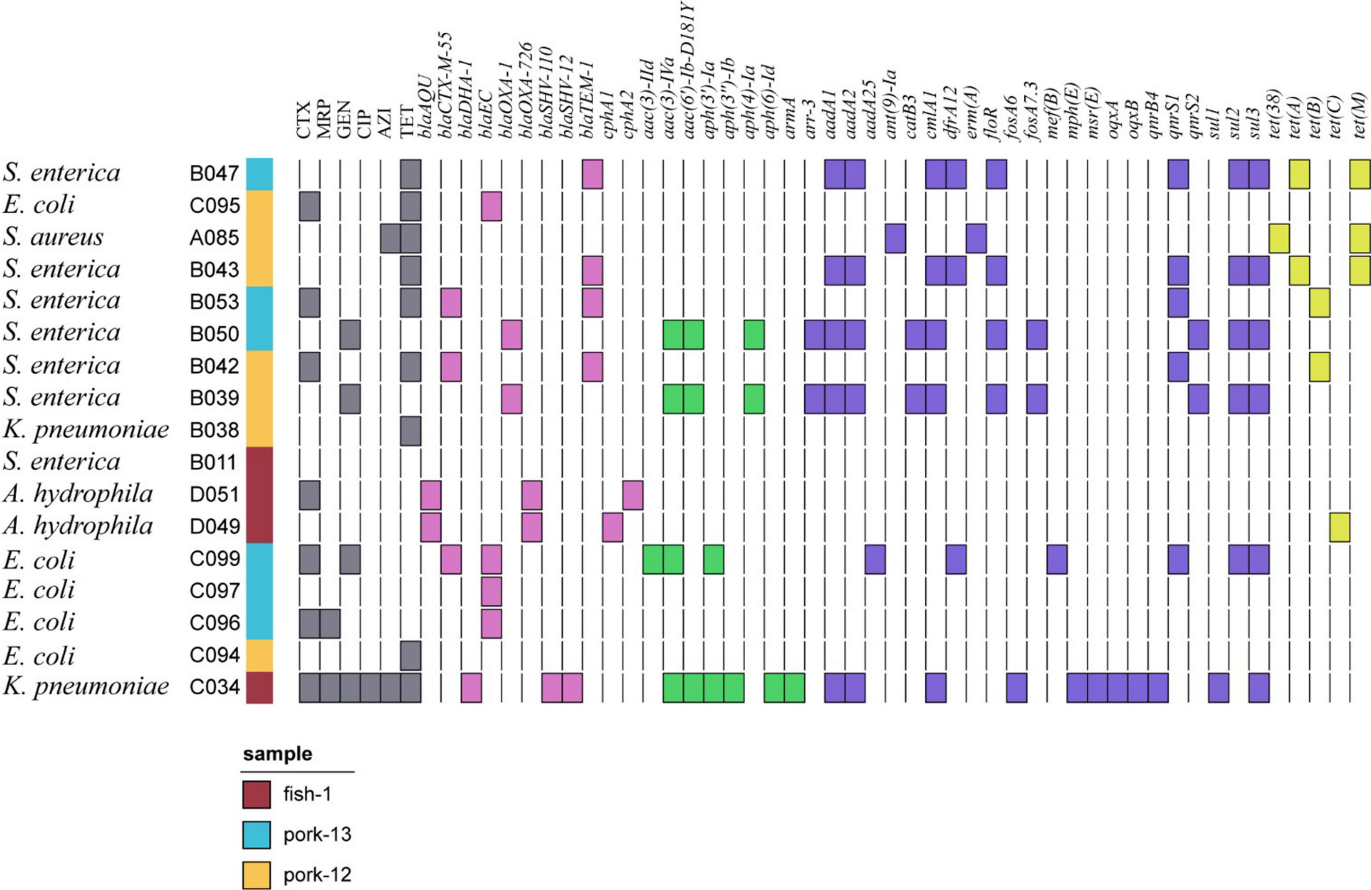
AMR phenotypes and ARGs located in the chromosome of meat-borne isolates. A total of 17 isolates recovered from three samples represented by three different colours are shown on the left. The corresponding AMR phenotypes and genotypes detectable in each bacterial chromosome are shown on the right. CTX, cefotaxime; MRP, meropenem; GEN, gentamicin; CIP, ciprofloxacin; AZI, azithromycin; TET, tetracycline.

To investigate the genetic relationship and transmission characteristics of AMR genes among different bacterial strains isolated from the same food sample, we conducted complete genome and plasmid sequence analysis of all bacterial strains isolated from three representative samples. A total of 17 strains were isolated from these 3 meat samples, namely pork-12, pork-13 and fish-1. Seven bacterial strains belonging to five different species were isolated from pork-12; six strains of two species were isolated from pork-13, and four strains that belonged to three species were isolated from fish-1. Phenotypically, all the 17 strains exhibited resistance towards tetracycline and cefotaxime, and among them, the *K. pneumoniae* strain C034 (acc no. SAMN40576002) was resistant to almost all the test drugs (Fig. 2). Results of the pairwise comparison between strains with or without plasmids suggested no sign of plasmid dissemination among strains of different species isolated from one or different samples. Apart from the resistance genes located in mobile elements, ARGs in the chromosomal regions of various isolates were identified by matching with the NCBI database; such chromosomal ARGs were found to encode resistance to different classes of antibiotics, such as beta-lactam, carbapenem, cephalosporin, quinolones and kanamycin (Table 4).

**Table 4.** ARGs in the chromosome of meat-borne strains and the genetic features of plasmids harboured by specific strains

Samples	Strains	Species	Chromosomal ARGs	Plasmids					Reference		
				Name	Size	G+C (%)	AGRs	Plasmid type	Acc no.	Query cover (%)	Per. identity (%)
Pork-12	A085	*S. aureus*	*ant(9)-Ia*, *erm(A)*, *tet(M)*, *tet(38)*	*\*							
	B038	*K. pneumoniae*	*fosA5*, *bla_SHV-1_*, *oqxA9*, *oqxB12*	pB038_15k	15393	45.5		ColRNAI_1	CP086763	100	99.68
	C094	*E. coli*	*bla_EC-15_*	pHK_C094_81k	81110	50.8	*tet(A)*, *sul2*, *floR*, *dfrA14*, *qnrS1*	IncX1_1, IncR_1	CP053570/MK656937	47/66	99.16/99.66
				pC094_50k	50847	50.7		IncFIA(HI1)_1_HI1, IncFIB(K)_1_Kpn3	CP090294	100	99.5
	C095	*E. coli*	*bla_EC_*	pC095_127k	127742	51.4		IncFIB(pHCM2)_1_pHCM2	CP090457	89	99.73
				pC095_28k	28886	46.8		IncR_1	CP074003	47	99.53
				pC095_79k	79529	51.8	*sul3*, *tet(A)*, *dfrA12*, *aadA2*, *cmlA1*, *aadA1*, *tet(M)*, *floR*, *sul2*, *bla_CTX-M-55_*, *qnrS1*	IncFIA(HI1)_1_HI1, IncX1_1	CP057369	49	99.72
	B039	*S*. Derby	*fosA7.3*, *qnrS2*, *arr-3*, *catB3*, *bla_OXA-1_*, *aac(6')-Ib-cr*, *floR*, *sul2*, *aph(4)-Ia*, *aac(3)-IVa*, *sul3*, *aadA1*, *cmlA1*, *aadA2*	pB039_16k	16885	49.4	*ANT(2'')-Ia*, *aadA2*, *sul1*		JQ418532	58	92.35
				pHK_B039_155k	155988	50.7	*bla_OXA-181_*, *qnrS1*, *fosA5*, *aph(6)-Id*, *aph(3'')-Ib*, *sul2*	IncA/C2_1	LC225353	85	99.669
	B042	*S. enterica* (4,5,12:i:-)	*bla_TEM-1_*, *tet(B)*, *qnrS1*, *bla_CTX-M-55_*	pB042_151k	151750	45.2	*ant(2'')-Ia*, *aadA2*, *sul1*	IncHI2_1, IncHI2A_1	CP050163	99	99.67
	B043	*S*. Rissen	*dfrA12*, *aadA2*, *cmlA1*, *aadA1*, *sul3*, *tet(A)*, *tet(M)*, *floR*, *sul2*, *bla_TEM-1_*, *qnrS1*	pB043_9k	9271	52.2		ColRNAI_1, Col440II_1	CP082690	99	98.32
Pork-13	C096	*E. coli*	*bla_EC-13_*	pHK_C096_186k	186788	47.4	*bla_CTX-M-98_*	IncHI1A_1, IncHI1B(R27)_1_R27, IncFIA(HI1)_1_HI1	CP051379	91	99.55
				pHK_C096_74k	73915	47.1	*bla_TEM-1_*	IncFIB(pHCM2)_1_pHCM2	CP099103	81	99.02
				pHK_C096_70k	70062	52.3	*bla_NDM-5_*, *ble_MBL_*	IncFII(pHN7A8)_1_pHN7A8	MN822125	89	99.7
				pC096_73k	73056	48.4		IncY_1	LC501468	79	97.07
	C097	*E. coli*	*bla_EC_*	pHK_C097_79k	79108	50.0	*bla_TEM-1_*, *floR*, *sul2*, *aadA1*, *qnrS1*	IncX1_1, IncFIA(HI1)_1_HI1, IncFIB(K)_1_Kpn3	AP027954	84	99.73
	C099	*E. coli*	*sul2*, *floR*, *dfrA12*, *aadA25*, *sul3*, *mef(B)*, *aph(3')-Ia*, *aac(3)-IId*, *bla_CTX-M-55_*, *qnrS1*, *bla_EC-18_*	pC099_90k	90077	50.5		IncFII_1	OK236218	93	99.47
	B047	*S*. Rissen	*dfrA12*, *aadA2*, *cmlA1*, *aadA1*, *sul3*, *tet(A)*, *tet(M)*, *floR*, *sul2*, *bla*_*TEM-1*_, *qnrS1*	pB047_9k	9275	52.3		ColRNAI_1, Col440II_1	CP082690	99	98.28
	B050	*S*. Derby	*qnrS2*, *arr-3*, *catB3*, *bla_OXA-1_*, *aac(6')-Ib-cr*, *floR*, *sul2*, *aph(4)-Ia*, *aac(3)-IVa*, *sul3*, *aadA1*, *cmlA1*, *aadA2*, *fosA7.3*	pB050_156k	156050	50.7	*sul2*, *aph(3'')-Ib*, *aph(6)-Id*, *fosA5*, *qnrS1*, *bla_OXA-181_*		CP027043	79	99.68
			*bla_TEM-1_*, *tet(B)*, *qnrS1*, *bla_CTX-M-55_*	pB050_90k	90570	47.4			CP046005	87	97.92
	B053	*S. enterica* (4,5,12:i:-)		pB053_151k	151750	45.2	*ant(2'')-Ia*, *aadA2*, *sul1*	IncHI2_1, IncHI2A_1	CP050163	99	99.67
Fish-1	C034	*K. pneumoniae*	*oqxA*, *oqxB*, *fosA6*, *bla_SHV-110_*, *bla_SHV-12_*, *qnrB4*, *bla_DHA-1_*, *sul1*, *armA*, *msr(E)*, *mph(E)*, *aph(3')-Ia*, *aph(3'')-Ib*, *aph(6)-Id*, *aph(4)-Ia*, *aac(3)-IVa*, *sul3*, *aadA1*, *cmlA1*, *aadA2*	pHK_C034_63k	63038	53.2	*floR*, *tet(D)*, *aac(6')-Ib-cr*, *arr-3*, *dfrA27*, *aadA16*, *sul1*	IncR_1, IncFIA(HI1)_1_HI1	CP021163	100	99.69
	D049	*A. hydrophila*	*bla_AQU_*, *tet(C)*, *bla_OXA-726_*, *cphA1*	\							
	D051	*A. hydrophila*	*bla_AQU-1_*, *bla_OXA-726_*, *cphA2*	\							
	B011	*S*. Stanley	*\*	\							

**Fig. 3.**
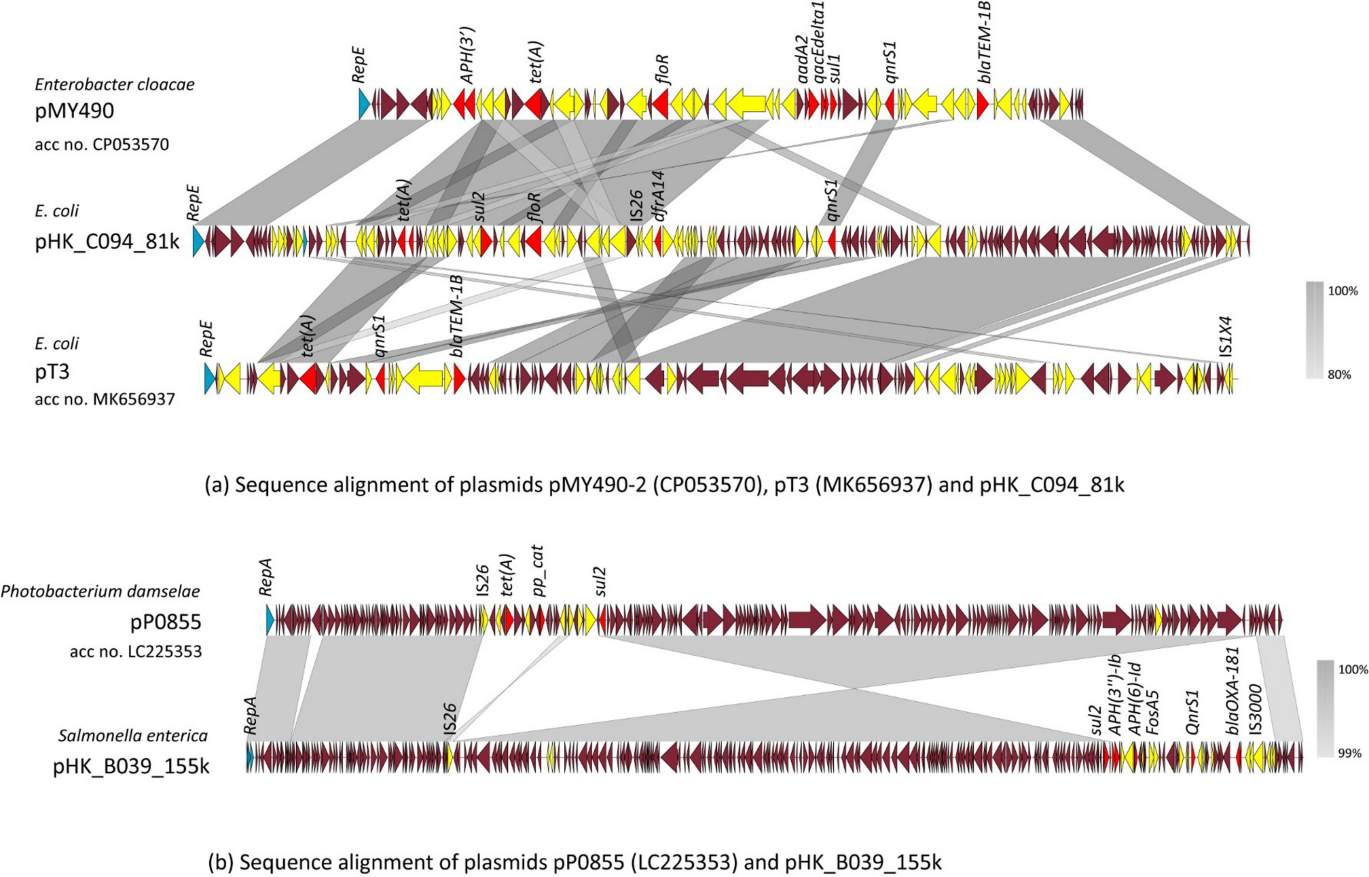
Schematic representation of the structure of two plasmids recovered from pork-12-derived strains. Results of sequence alignments between (**a**) *Enterobacter cloacae* plasmid pMY490-2 (CP053570), *E. coli* plasmid pT3 (MK656937) and pHK_C094_81k and (**b**) *P. damselae* plasmid pP0855 (LC225353) and pHK_B039_155k are shown. CDSs without labels represent hypothetical proteins. The shadow parallelograms denote genetic regions that exhibit sequence homology. Light shadow denotes regions with a lower level of sequence identity. Arrows indicate CDSs, with arrowheads indicating the direction of transcription: red, antibiotic resistance-encoding genes; yellow, mobile elements; blue, replication protein-encoding genes; deep burgundy, maintenance/stability genes, or genes that encode hypothetical proteins.

Bacterial strains isolated from three representative samples were analysed in detail. The first sample, pork-12, was collected from a wet market located in northern Hong Kong. Nine bacterial strains were isolated in the raw meat, including one Gram-positive isolate (*S. aureus* A085, acc no. SAMN40575989) and eight Gram-negative strains, of which seven strains, except one *Aeromonas hydrophila*, were sequenced. Alignment of plasmids showed that each of the seven Gram-negative bacterial strains carried at least one plasmid, most of which were highly analogous with previously reported data, but a few were recognized as novel (Fig. 3). The *S. aureus* strain A085 was found to carry a number of ARGs in the chromosome, but no plasmid was detectable. One of the Gram-negative strains, *K. pneumoniae* strain B038 (acc no. SAMN40575990), carried a few intrinsic ARGs and a small ColRNAI_1 plasmid that did not contain any ARGs and was structurally similar to a plasmid pAZS099-2 (GenBank: CP086763). *E. coli* strain C094 (acc no. SAMN40575991) was sensitive to most antimicrobials including cefotaxime and meropenem. This strain carried the *bla_EC-15_* gene and a new plasmid, pHK_C094_81 k, which appears to be a fusion plasmid formed by integration of specific segments of the plasmids pMY490 and pT3, with a low coverage of, respectively, 47 and 66%, but >99% identity to these two plasmids. The resistance gene *dfrA14* found in this plasmid was probably derived from a newly inserted genetic fragment as neither of the two reference plasmids bears this gene. The *E. coli* strain C095 (acc no. SAMN40575992) was found to harbour a *bla*_EC_ gene located on chromosome, as well as three plasmids, namely pC095_127k, pC095_28k and pC095_79k, and the plasmids pC095_127k and pC095_28k did not carry any ARG and exhibited a certain degree of sequence homology with plasmids pB379_2 (89%, GenBank: CP090457) and pYLPM4b (47%, GenBank: CP074003), respectively. Plasmid, pC095_79k, was found to carry multiple ARGs including *bla*_CTX-M-55_; it is likely that this plasmid formed by fusion between the IncFIA(HI1)_1_HI1 plasmid and an IncX1_1 plasmid. In the genome of *S*. Derby B039 (acc no. SAMN40575993), there were a variety of chromosomal genes including *bla_OXA-1_*, *qnrS2*, *arr-3*, *catB3*, *aac(6')-Ib-cr*, *aph(4)-Ia*, *aac(3)-IVa* and *cmlA1*. This strain also carried an MDR plasmid, pHK_B039_155k, which exhibited 85% homology with the plasmid pP0855 from a *Photobacterium damselae* strain but differed dramatically in the MDR region that carried different types of ARGs, as very few ARGs were found in pHK_B039_155k. In another two *Salmonella* strains, B042 (acc no. SAMN40575994) and B043 (acc no. SAMN40575995), multiple ARGs including *bla*_CTX-M-55_ were detectable in the chromosome. Each of these two strains also carried one plasmid that was highly analogous with (KPC-2)_IncHI2 (GenBank: CP050163) and pN18S0406-4 (GenBank: CP082690), respectively.

**Fig. 4.**
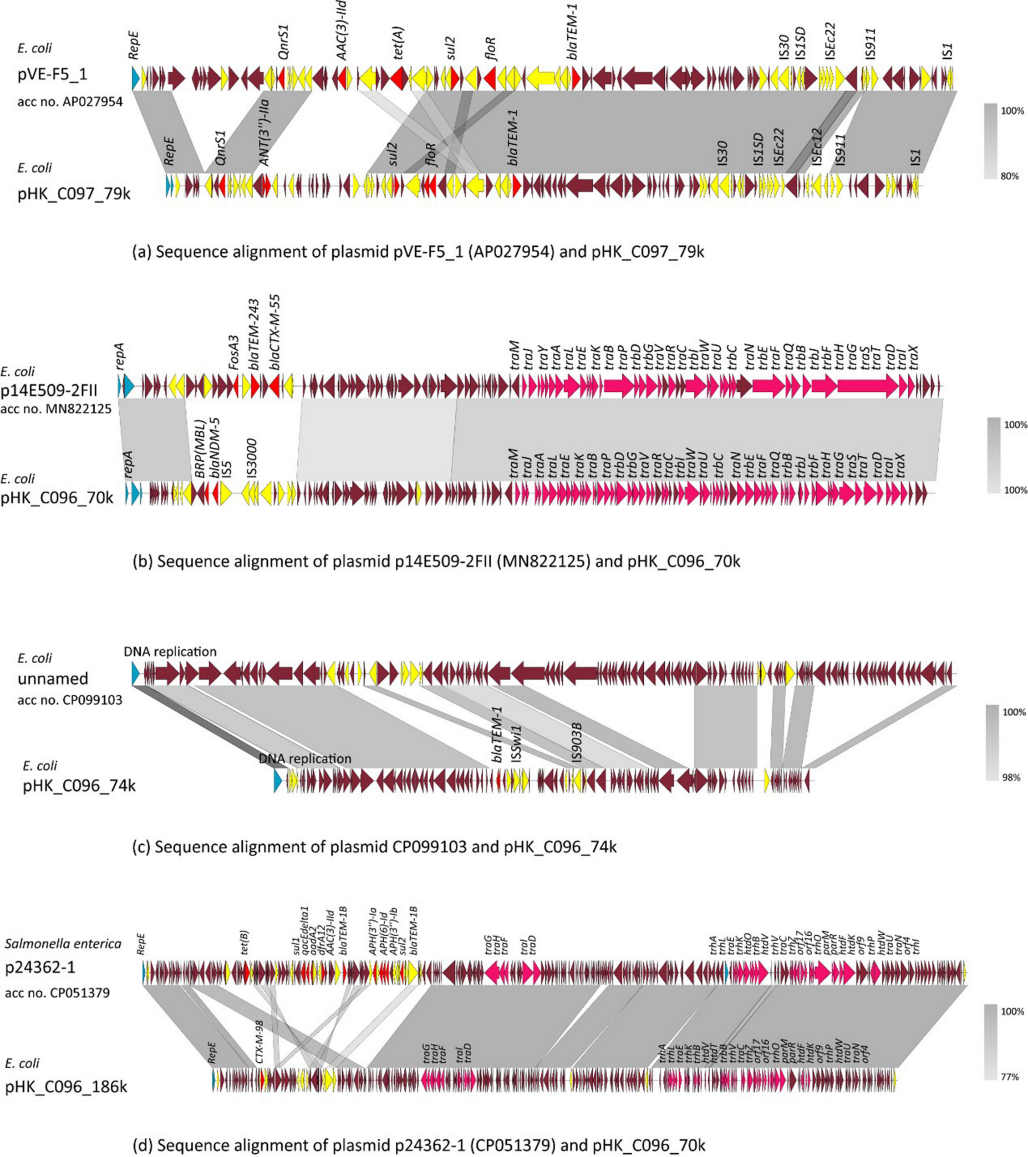
Schematic representation of the structure of four plasmids recovered from pork-13-derived isolates. Results of sequence alignments between (**a**) *E. coli* plasmid pVE-F5_1 (AP027954) and pHK_C097_79k, (**b**) *E. coli* plasmid p14E509-2FII (MN822125) and pHK_C096_70k, (**c**) *E. coli* plasmid CP099103 and pHK_C096_74k and (**d**) *S. enterica* plasmid p24362-1 (CP051379) and pHK_C096_186k are shown. CDSs without labels represent hypothetical proteins. The shadow parallelograms denote genetic regions that exhibit sequence homology. Light shadow denotes regions with a lower level of sequence identity. Arrows indicate CDSs, with arrowheads indicating the direction of transcription: red, antibiotic resistance-encoding genes; yellow, mobile elements; blue, replication protein-encoding genes; pink, genes associated with the *tra* loci; deep burgundy, maintenance/stability genes, or genes that encode hypothetical proteins.

The second sample, pork-13, was collected from an eastern Hong Kong market, from which three *E. coli* strains and three *Salmonella* strains were isolated. *E. coli* strain C097 (acc no. SAMN40575997) remained sensitive to most antimicrobial drugs including the cephalosporins, except that low-level resistance to ciprofloxacin was detected. The gene *bla_EC_* could be detected in the genome of C097. A plasmid – pHK_C097_79k – was recovered from this strain and found to exhibit a coverage rate of 84% with the plasmid pVE-F5_1 (Fig. 4), which was recovered from a colistin-resistant *E. coli* isolated from Vietnam; pHK_C097_79k also carried five AMR genes including *bla_TEM-1_*, as well as several mobile elements, such as *IS*1 and *IS*Ec22. With a size of 79,108 bp, the plasmid type of pHK_C097_79k was presented as IncX1_1/IncFIA(HI1)_1_HI1/IncFIB(K)_1_Kpn3 (Table 4).

Another *E. coli* isolate, C096 (acc no. SAMN40575996), also carried the beta-lactamase-encoding gene *bla*_EC-13_ in the chromosome. C096 exhibited broad-spectrum resistance to the third-generation cephalosporins cefotaxime and ceftazidime, and the carbapenem antibiotic of meropenem. Consistently, three MDR plasmids which carried the ARGs *bla*_CTX-M-98_, *bla_TEM-1_*, and *bla_NDM-5_*/*ble_MBL_*, respectively, could be detected in this strain. The size of these three plasmids, namely pHK_C096_186k, pHK_C096_74k and pHK_C096_70k, was 186,788, 73,915 and 70,062 bp, with a G+C content of 47.4 mol% and 47.1–52.3 mol%, respectively. Plasmid pHK_C096_186k was shown to be homologous to plasmid p24362-1 [21], which was recovered from an *S. enterica* strain previously. However, the MDR region in pHK_C096_186k was structurally very different from that of p24362-1 [21], suggesting that evolution of this type of plasmid occurs in different organisms. A *bla_CTX-M-98_* gene was found located in the MDR region of pHK_C096_186k. Plasmid pHK_C096_70k was an IncFII plasmid containing a *bla*_NDM-5_ gene-bearing IS3000 mobile element. It exhibited a high degree of sequence homology with plasmid p14E509-2FII from an *E. coli* strain from China, except for the MDR region. The MDR region in plasmid p14E509-2FII contained a *bla*_CTX-M-55_ gene rather than a *bla*_NDM-5_ gene, suggesting that this plasmid actively evolves. It is also predicted to be a conjugative plasmid which carried the *tra* locus, thereby enabling it to transfer the carbapenemase gene *bla_NDM-5_* to other antibiotic-susceptible organisms. Plasmid pHK_C096_74k exhibited a relatively high degree of homology (81%) to an unnamed plasmid (CP099103.1) from *E. coli* isolated from a pooled sediment sample collected from the floor of a pig farm in the UK. Unlike the unnamed plasmid that did not carry any AMR gene, pHK_C096_74k carried a *bla*_TEM-1_ gene. *S*. Rissen strain B047 (acc no. SAMN40575999) carried multiple ARGs which were located in the chromosome, as well as a small 9k plasmid; *S*. Derby strain B050 (acc no. SAMN40576000) carried multiple ARGs that were located in the chromosome, as well as two plasmids, namely pB050_156k and pB050_90k, both of which exhibited over 80% homology with previously reported plasmids; *S*. enterica (4,5,12:i:-) strain B053 (acc no. SAMN40576001) carried multiple ARGs, including a *bla*_CTX-M-55_ gene in the chromosome and a 151k plasmid which has been reported previously (Table 4).

**Fig. 5.**
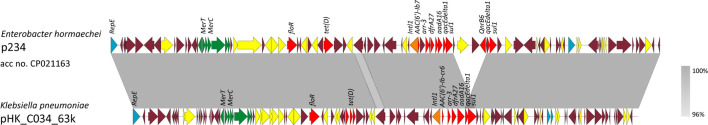
Schematic representation of the structure of plasmid pHK_C034_63K of a fish-1-derived *K. pneumoniae* strain. The result of sequence alignments between *E. hormaechei* plasmid p234 (CP021163) and pHK_C034_63k is shown. CDSs without labels represent hypothetical proteins. The shadow parallelograms denote genetic regions that exhibit sequence homology. Light shadow denotes regions with a lower level of sequence identity. Arrows indicate CDSs, with arrowheads indicating the direction of transcription: red, antibiotic resistance-encoding genes; yellow, mobile elements; blue, replication protein-encoding genes; orange, integrase gene; green, genes associated with silver and heavy metal resistance; deep burgundy, maintenance/stability genes, or genes that encode hypothetical proteins.

Lastly, the third sample, fish-1, which was purchased from a market in the northwestern part of Hong Kong, was found to harbour one *K. pneumoniae* strain (C034), two *Aeromonas* strains, one *S. enterica* Stanley strain (B011, acc no. SAMN40576005) and one *E. faecalis* strain (A049). *K. pneumoniae* strain C034 exhibited the widest resistance spectrum, with a large number of ARGs being identified in the chromosome [*bla_DHA-1_*, *bla_SHV-110_*, *bla_SHV-12_*, *aph(3')-Ia*, *aph(3'')-Ib*, *aph(6)-Id*, *aph(4)-Ia* and *aac(3)-Iva*]. In addition, it also carried a plasmid, pHK_C034_63k, with a size of 63,038 bp and 53.2 mol% of G+C content. This plasmid was found to contain multiple aminoglycoside resistance genes [*aac(6')-Ib-cr6*, *aadA16*] (Fig. 5). Several types of mobile genetic elements, including MGE IS*1006*, IS*Vsa3*, IS*26*, IS*6100*, IS*15DI* and IS*1A*, were identified in pHK_C034_63k, the plasmid type of which was likely to be IncR_1/IncFIA(HI1)_1_HI1 (Table 4). The large number of ARGs detected is consistent with the multidrug resistance phenotype observable in this strain. Alignment between pHK_C034_63k and plasmid p234 harboured by the organism *Enterobacter hormaechei* (query coverage 100%, per. identity 99.7%) showed near-complete synteny. The only divergent region was a ~4 kb segment absent in pHK_C034_63k, corresponding to a cluster of hypothetical proteins and a partial mobile element in p234. No ARGs or known functional modules were located in this missing region. The other three strains, namely *A. hydrophila* D049 (acc no. SAMN40576003), *A. hydrophila* D051 (acc no. SAMN40576004) and *S. Stanley* B011, did not carry any plasmid, but a few ARGs were found in the chromosome.

## Discussion

This study provides a focused characterization of plasmid structures and ARG contexts in Hong Kong wet-market meat using long-read sequencing. The detection of several previously unreported plasmid fusion structures and distinct ARG combinations highlights regional variation in AMR dissemination dynamics and contributes new genomic evidence specific to Hong Kong’s retail food environment. Among the 236 isolates recovered from 95 raw meats in this survey, *E. coli* was the most commonly isolated bacterial species in most samples, and pork was the most favourable habitat of this species. Other species generally exhibited low-level prevalence in most of the food samples. *B. cereus* strains were identified in 6 out of the 95 samples as they habitually contaminate rice and dairy products and may cause diarrhoea or emetic syndrome [22,23]. The main source of 16 *S. enterica* strains was also pork (14 out of 16) rather than chicken. From a global viewpoint, reports from the CDC and EFSA have confirmed that pig meat was most frequently associated with *S. enterica* serovar Typhimurium [24,26]. Several *K. pneumoniae* and *E. faecalis* isolates, accompanied by several other unique species, colonized a small proportion of meats, which is likely underrepresented as they were not intended to be included in the isolation process. Nonetheless, previous reports have illustrated the potential by which *E. faecalis* transfers genetic elements to human strains following consumption of contaminated meat, and that *E. faecalis* and *E. faecium* were found in 69.5 and 11.3% of the red meat samples (fresh beef and pork), respectively [27,28]. The prevalence of *Aeromonas* spp. in fish and shrimps (40 and 60%) was quite high when compared to another study focused on Norwegian ready-to-eat seafood, where the maximum rate appeared to be 17% in retail sushi [29]. Although the sample size in this study was not large enough, it is reasonable to speculate that this discrepancy is due to the fact that the warm weather in Hong Kong and its location in the sub-tropical zone favour the survival and circulation of various pathogenic organisms. The sample size (95 raw meat samples) provides a snapshot rather than population-level estimates. The different numbers of food types reflected availability and sales volume in local wet markets during sampling, rather than deliberate stratified design. As the study was exploratory, results should not be generalized to the entire Hong Kong food supply. A larger, longitudinal dataset would be needed to assess temporal or territory-wide epidemiological trends.

AMR is one of the most important concerns in current efforts to combat foodborne bacterial infections. A Canadian study showed that *E. coli* strains recovered from broiler chicken exhibited a high rate of resistance to aminoglycosides, β-lactams and tetracyclines, yet only 0.2% of such strains were resistant to ciprofloxacin. The percentage of *Salmonella* isolates that were resistant to β-lactams, macrolides, quinolones and tetracyclines was similar but generally lower than that of *E. coli* [30]. In a study in TN, USA, bulk tank milk, faeces and soil samples were collected in four dairy cattle farms, and antimicrobial tests of bacteria isolated from such samples confirmed that *E. coli* exhibited the highest rate of resistance (73.9%) to TET, followed by TGC (cefotaxime) (20.5%) and NAL (nalidixic acid) (8%), although the proportion of AMR *E. coli* varied by farms and samples [31]. In our study, meat-borne *E. coli* generally exhibited higher rates of resistance to cefotaxime (48.9%, 64 out of 131) but a comparatively modest rate of resistance to the other two β-lactams, meropenem and ceftazidime. Previously, *K. pneumoniae* was detected in raw chicken and pork liver at a frequency of 27% (16 of 60) in Singapore; in the same study, half of the *K. pneumoniae* strains that exhibited the MDR phenotype isolates were isolated from meat samples (5 out of 10) [32]. An even higher rate of isolation (81.8%) of *K. pneumoniae* strains in Greek meat products was reported, with *bla_NDM_* (61.5%) and *bla_OXA-48_*-like (30.8%) genes being the most frequently detected resistance genes [33]. In this project, we also studied the genetic features of 17 bacterial isolates recovered from 3 different meat samples, with a focus on the genetic context of the plasmids that harboured resistance genes and mobile elements recoverable from these strains. Bioinformatic analysis suggested that, among the strains recovered from samples gathered from distinct local markets and organisms isolated from the same food sample, evidence of direct plasmid transmission could not be obtained. Instead, six new plasmids with unique structures were detectable among such strains. Among the three *E. coli* strains, we recovered a total of five plasmids, among which the *bla_TEM-1_*-bearing pHK_C097_79k did not match with any plasmid replicon type in the PlasmidFinder database [34]. A number of insertion elements including IS*30*, IS*1*, IS*66* and IS*3* were also found in this plasmid; such elements were found to have acquired a known copy-paste mechanism that generates a transient double‐strand circular DNA intermediate [35,38]. Moreover, another three plasmids carried by the same *E. coli* strain (C096) were found to belong to the IncHI1A, IncFIB and IncFII types, respectively; these plasmids contained various β-lactamase genes. Similarly, another study in China described a plasmid, pHeBE7, which carried the *bla_CTX-M-98b_*, *bla_TEM-1_*, *rmtB* and *traT* genes and could spread to geographically diverse regions through human or food animals [39]. In addition, the *bla_NDM-5_* and *tet(X4*) genes co-harboured by the clinically isolated *E. coli* ST648 strain were found to encode resistance to carbapenem and tigecycline, respectively, indicating that plasmid-borne resistance genes in various superbugs can pose a serious threat to public health [40]. In Singapore, researchers reported the complete nucleotide sequence of a transferable plasmid harbouring the *bla_TEM-176_* and *mcr-5.1* genes in an *E. coli* strain isolated from ready-to-eat chicken rice [41]. The *Salmonella* plasmid pHK_B039_155k identified in this work was categorized as the IncA/C2 type and found to carry an array of ARGs [*bla*_OXA-181_, *qnrS1*, *fosA5*, *aph(6)-Id*, *aph(3'')-Ib* and *sul2*] that encode resistance to five classes of antimicrobials, but phenotypically, the strain was resistant to gentamicin only. Factors that regulate the expression of plasmid-borne antibiotic resistance genes in bacterial pathogens need to be identified in future experiments. Also, because only isolates from Hong Kong Island were subjected to long-read sequencing, territorial comparison (HK vs. Kowloon vs. NT) is limited. Inclusion of additional sequenced isolates from other districts would improve the geographic resolution of plasmid and ARG distribution.

In conclusion, we have conducted a general surveillance of foodborne pathogens collected in Hong Kong markets and depicted the AMR profiles of selected bacterial strains. Several plasmids harboured ARGs and mobile elements capable of mediating horizontal transfer. Although this study did not experimentally assess transferability, the structures identified suggest plausible vehicles for ARG dissemination, warranting future functional validation.

## Abbreviations

AMR: antimicrobial resistance
ARGs: AMR-encoding genes
CLSI: Clinical and Laboratory Standards Institute
CRE: carbapenem-resistant *Enterobacteriaceae*
MDR: multidrug-resistant
MICs: minimal inhibitory concentrations
